# Combined Effect of Temperature and Relative Humidity on the Survival of *Salmonella* Isolates on Stainless Steel Coupons

**DOI:** 10.3390/ijerph19020909

**Published:** 2022-01-14

**Authors:** Amreen Bashir, Peter A. Lambert, Yvonne Stedman, Anthony C. Hilton

**Affiliations:** 1 College of Health & Life Sciences, Aston University, Birmingham B4 7ET, UK; p.a.lambert@aston.ac.uk (P.A.L.); a.c.hilton@aston.ac.uk (A.C.H.); 2 Salvus Food Consulting Ltd, P75 VP83 County Cork, Ireland; yvonne@salvus.ie

**Keywords:** *Salmonella*, survival, temperature, humidity, food-manufacturing, stainless steel

## Abstract

The survival on stainless steel of ten *Salmonella* isolates from food factory, clinical and veterinary sources was investigated. Stainless steel coupons inoculated with *Salmonella* were dried and stored at a range of temperatures and relative humidity (RH) levels representing factory conditions. Viability was determined from 1 to 22 days. Survival curves obtained for most isolates and storage conditions displayed exponential inactivation described by a log-linear model. Survival was affected by environmental temperatures and RH with decimal reduction times (DRTs) ranging from <1 day to 18 days. At 25 °C/15% RH, all isolates survived at levels of 10^3^ to 10^5^ cfu for >22 days. Furthermore, temperatures and RH independently influenced survival on stainless steel; increasing temperatures between 10 °C and 37 °C and increasing RH levels from 30–70% both decreased the DRT values. Survival curves displaying a shoulder followed by exponential death were obtained for three isolates at 10 °C/70% RH. Inactivation kinetics for these were described by modified Weibull models, suggesting that cumulative injury occurs before cellular inactivation. This study highlights the need to control temperature and RH to limit microbial persistence in the food manufacturing environment, particularly during the factory shut-down period for cleaning when higher temperature/humidity levels could be introduced.

## 1. Introduction

*Salmonella* are important foodborne pathogens that are able to cause cross-contamination of food products and are a leading cause of foodborne illness worldwide [1]. As a ubiquitous organism, *Salmonella* occurs naturally in the environment and can pass via multiple routes in the food chain from producers to consumers. *Salmonella* contamination has been implicated in a range of food products, including poultry, fish eggs, dairy, vegetables and dry foods [2,3]. For many years, food products have been preserved via drying, as low moisture foods have an increased shelf life and are considered stable for years [4]. Despite the common misconception regarding the contamination of low water activity produce, these products are subject to microbial contamination and organisms, such as *Salmonella*, may survive [3,4,5]. Notable outbreaks associated with low-moisture foods have been reported, including those associated with cereal, chocolate, dried milk products, salami and pet food [6,7,8,9]. The food manufacturing processes associated with some of these included thermal inactivation, suggesting that cross-contamination is likely to occur post-treatment with *Salmonella* that have become established in the manufacturing environment.

Procedures are in place to minimise the movement of staff and materials between pre-processed and processed areas within the factory, including designated separate routes of entry and movement of goods [10,11]. Despite these measures, the finished goods environment still provides a niche for the survival of these microorganisms in the finished products. Although machinery and food processing equipment are designed to be accessible to cleaning and disinfection and prevent cross-contamination, they still offer an opportunity for microbes to harbour and provide a source of cross-contamination [10,12]. In addition, with age and poor maintenance, walls with cracks, damaged machinery, water pipes and gutters all pose a risk of cross-contamination due to the accumulation of microorganisms [13].

Various studies have been conducted on surfaces found in food processing plants, including stainless steel, granite and plastic [14,15]. Stainless steel is commonly used in factories for working surfaces, pipes, tanks and machinery. It has many properties which make it an ideal surface; for example, it is resistant to corrosion as it is protected by a layer of naturally occurring chromium oxide on the surface. Stainless steel is also inert and does not contribute any taint to food products making contact with the equipment and it can withstand low and high temperatures; most importantly, it is also relatively easy to clean [16].

Temperature and humidity levels fluctuate in food manufacturing environments due to the different environmental conditions maintained in the manufacturing, processing and packaging zones. Weather conditions can also influence the temperature and humidity inside production sites. Resident factory isolates are those that are adapted to the environment and have been repeatedly isolated over a period of time. Modelling dry and humid conditions that may be found in a food factory setting, Habimana et al. [14] investigated the survival of *Salmonella* on stainless steel coupons at 12°C with high humidity (85%) and at 30°C with low RH (35%). The results showed a marked decrease in *Salmonella* survival with a low temperature and high humidity combination, emphasising the need to further study the interaction between these two parameters. Another study which investigated the survival of *Salmonella* strains desiccated on stainless steel and stored at 25 °C with 33% humidity showed that they could survive for a duration of at least 30 days, however survival was not linked to serotype; the highest and lowest survival was observed for *Salmonella* Typhimurium [17].

It is essential to understand how *Salmonella* is introduced and persists in food manufacturing environments. The aim of this study was to identify the combined effect of temperature and humidity on the survival on stainless steel of a range of *Salmonella* isolates from factory and veterinary sources, clinical isolates from outbreaks associated with pet food and reference strains.

## 2. Material and Methods

### 2.1. Bacterial Strains

A panel of ten *Salmonella* was created comprising isolates known to be persistent in the pet food factory environment, veterinary and well-characterised reference strains (Table 1). The factory isolates originated from environmental swabs collected at two pet food manufacturing sites producing dry complete pet food and wet food in tins and pouches. Environmental swabs were collected daily at 20–40 sample points. Persistent strains were defined as those isolated repeatedly from designated sample points within the factory on more than eight independent occasions. All swab locations were mapped and documented as part of the factory master sanitation program.

Swabbing was conducted using sterile, cellulose sponge swabs pre-moistened with a 10 mL sodium thiosulphate buffer and containing neutralizers of Tween 80 and lecithin (Product code: TS/15-A-95, TSC Ltd., Lancashire, UK). Pet food factory isolates included *S*. Senftenberg, *S*. Livingstone *S*. Kedougou, *S*. Montevideo and *S*. Schwarzengrund. As far as possible, isolates from the different environments were serotype matched. Veterinary strains from canine isolates were obtained from the Veterinary Laboratory Agency, Surrey, UK and included *S*. Senftenberg and *S*. Schwarzengrund. *S.* Schwarzengrund caused an outbreak associated with pet food in 2008 [7] and the *S.* Schwarzengrund (FSL S5-458) is the American clinical isolate which was isolated from patients during the outbreak. The heat resistant strain *S.* Senftenberg 775W is well-documented and, unlike other strains, it is a non-hydrogen sulphide producer. Globally, *S.* Senftenberg 775W (ATCC 43845) is not a major cause of salmonellosis but outbreaks are commonly associated with contaminated poultry and plant derivative food [18]. *S.* Typhimurium wild-type strain SL1344 was included as a well-characterised strain and the literature shows that the serotypes Typhimurium and Enteritidis are the leading causes of *Salmonella* disease [1,2]. All were stored on Microbank beads (Fisher Scientific, Loughborough, UK) and maintained at −80 °C until required. Table 1 shows the panel of isolates selected for the investigations detailing the origin of the isolate. Serotype-matched clinical and veterinary isolates were sourced for the pet food factory isolates of *S*. Senftenberg and *S*. Schwarzengrund to balance serotypes.

### 2.2. Pet Food Factory Environmental Monitoring

The relative humidity (RH) and ambient temperature of a factory producing heat-extruded products subjected to ambient cooling were monitored every 10 min for two months (May–July) using a Hygropalm-HP21 data logger (Rotronic, West Sussex, UK). The manufacturing cycle was four-day production, followed by three-day shutdown. These environmental data were used to select the combinations of RH and temperature explored in the survival studies (listed in Table 2). To maintain selected RH conditions, plastic airtight containers (Azpack, UK) measuring 280 × 160 × 103 mm were used with saturated salts added to control the humidity and placed into incubators at the desired temperature (±0.5 °C). Each container was fitted with a thermohygrometer (Cole-Parmer) to monitor the RH and temperature of each container.

The initial experiments included the panel of ten *Salmonella* isolates and were conducted at the following temperatures and relative humidity levels: 37 °C/20% RH, 25 °C/15% RH and 10 °C/70% RH. These were chosen to reflect environmental conditions measured in different regions of a manufacturing unit. To further investigate the interplay between temperature and relative humidity, subsequent experiments focussed on the three serotype-matched *S.* Schwarzengrund isolates associated with the 2008 pet food outbreak [7]. The survival conditions comprised all combinations of three temperatures (10 °C, 25 °C and 37 °C) with three RH levels (30%, 50% and 70% RH).

### 2.3. Survival Experiment

A 0.9 mm thick stainless steel sheet (Metals4U Wetherby, West Yorkshire, UK) was cut into 10 × 10 mm square coupons which were heat sterilised by autoclaving at 121 °C for 15 min. Bacterial strains were grown in 30 mL of nutrient broth (Oxoid CM0001, Fisher Scientific, Thermo, UK) for 18 h at 37 °C in an orbital shaker. Heat-sterilised coupons were placed in sterile petri dishes. Each coupon was inoculated with 10 μL of the overnight culture containing 10^8^ cfu/mL and allowed to dry in a static incubator for 60 min at 37 °C. Following drying, the coupons were placed in plastic airtight containers containing saturated salts to maintain the desired RH and held at the designated temperatures in static incubators. At the selected time points of 0, 2, 7, 10, 14 and 22 days, coupons were removed from the petri dishes using sterilised forceps and the number of viable bacteria was determined. This was achieved by adding each coupon to 10 mL of 0.085% *w*/*v* saline solution and mixing by vortexing for 2 min. Preliminary experiments showed that this procedure was effective in dislodging >99% of all organisms from the steel coupons. This was confirmed by direct examination of the coupon surfaces before and after vortexing using epifluorescence microscopy with SYTO9/propidium iodide staining. Ten-fold dilutions were performed by adding 0.1 mL of vortexed suspensions to 0.9 mL of saline solution and mixing, further serial 10-fold dilutions were made until a final 10^−4^ dilution was achieved. The surface of nutrient agar plates (Oxoid CM0003, Fisher Scientific, Thermo, UK) were inoculated by spreading 0.1 mL of each diluted suspension. Following 18–24 h incubation at 37 °C, the colony counts were recorded. Colonial appearance was monitored on all plates at specified time intervals throughout the 22 day duration of the experiments to confirm that contamination of the coupons with other organisms did not occur.

### 2.4. Data Preparation and Analysis

Survival of isolates on the stainless steel surfaces under various conditions of temperature and RH was presented in the form of graphs of log_10_ viable colony forming units (cfu) per coupon against time. Viable counts were taken over 22 days. Separate coupons were used for each time point and viable counts are presented for the mean and standard deviation of three individual replicate coupons at each time point. The survivor curves were categorised according to the different shapes described and numbered by Geeraerd et al. [19] using the Geeraerd and Van Impe model fitting tool, GInaFit v1.6. Decimal reduction times (DRT) were calculated as −1/slope determined by log-linear regression (i.e., linear regression of the log_10_ cfu per coupon vs. time data) using GraphPad Prism version 8.1.0 for Windows (GraphPad Software, La Jolla, CA, USA). DRT is presented with the associated temperature as a subscript in the results section, e.g., D_10_, D_25_ and D_37_. In cases where the survivor curves did not follow a log-linear pattern the GInaFit software was used to assign alternative model structures indicating the underlying mechanisms involved [20,21]. For these isolates, delta values derived from the modified Weibull model of Albert and Mafart [22], indicating the time for the first decimal reduction in survivors, were also calculated. The parameters for modified Weibull models of non-linear survivor curves derived from Albert and Mafart [22] using GInaFiT [19] are provided in Supplementary Materials, Table S1.

## 3. Results

**Figure 1:**
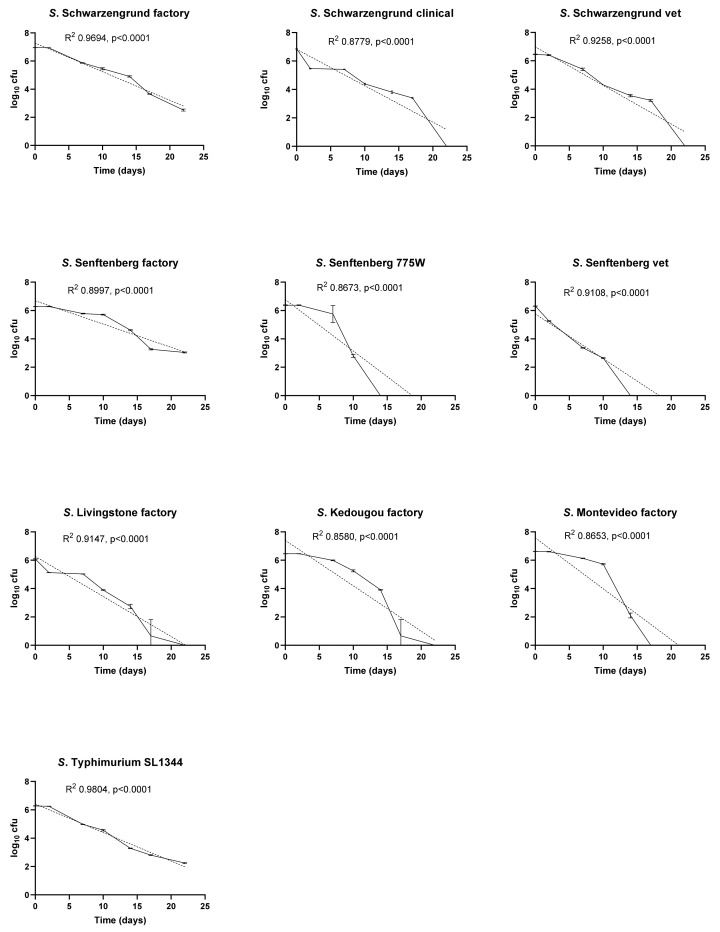
Survival of ten *Salmonella* isolates on stainless steel at 10 °C, 70% RH. Viable counts are shown as log_10_ cfu/coupon at each time point. Results are plotted as means of triplicate experiments with bars showing standard deviation. Best-fit lines determined by log-linear regression are included for each isolate with R^2^ and *p* values.

**Figure 2:**
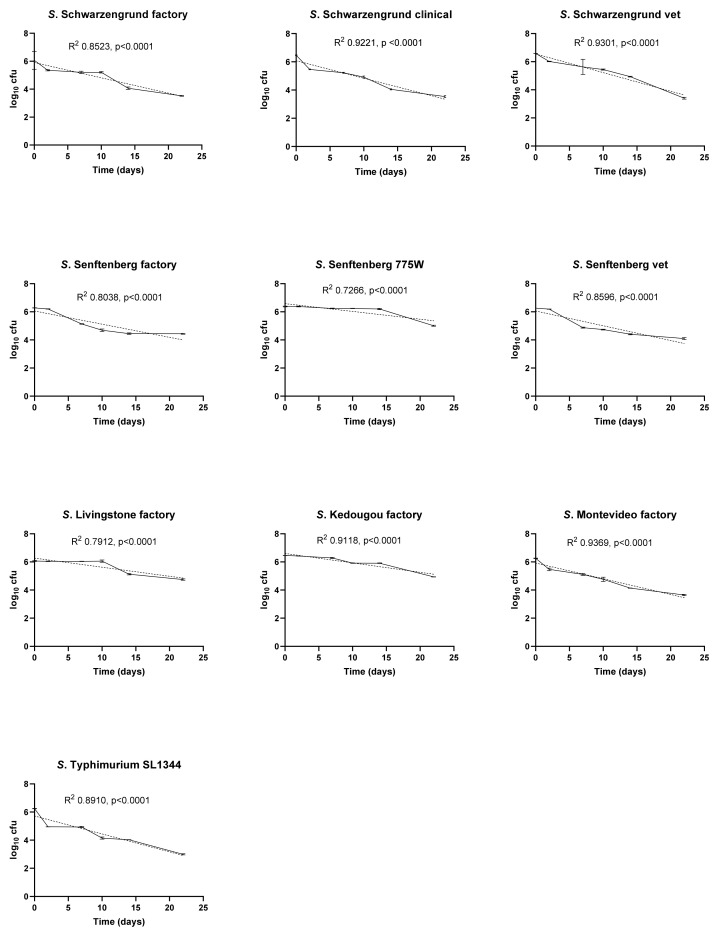
Survival of *Salmonella* isolates on stainless steel at 25 °C, 15% RH. Viable counts are shown as log_10_ cfu/coupon at each time point. Results are plotted as means of triplicate experiments with bars showing standard deviation. Best-fit lines determined by log-linear regression are included for each isolate with R^2^ and *p* values.

**Figure 3:**
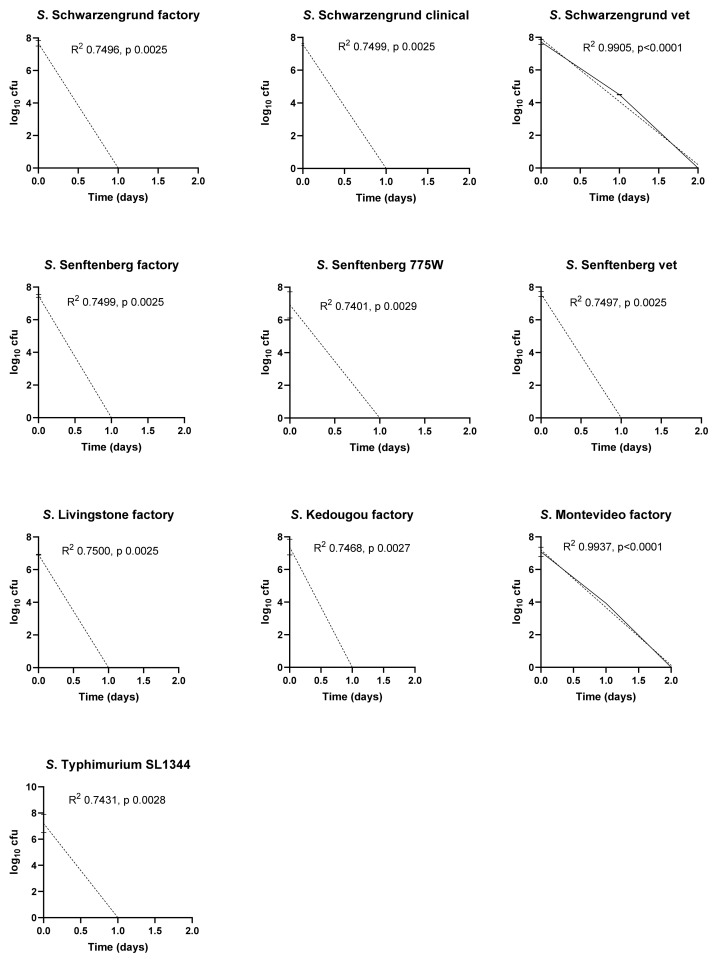
Survival of *Salmonella* isolates on stainless steel at 37 °C, 20% RH. Viable counts are shown as log_10_ cfu/coupon at each time point. Results are plotted as means of triplicate experiments with bars showing standard deviation. Best-fit lines determined by log-linear regression are included for each isolate with R^2^ and *p* values.

The initial experiments investigated the effect of temperature and RH on the survival of a panel of ten *Salmonella* isolates (Figure 1, Figure 2 and Figure 3). Under all temperature and relative humidity conditions, most isolates displayed log-linear survivor curves, classified as ‘linear’ by Geeraerd et al. [19], indicating first order kinetics for cell death. DRT values for each isolate under all conditions calculated from the slopes of the survivor curves in Figure 1, Figure 2 and Figure 3 are listed in Table 3. At 10 °C/70% RH, the survivor curves of three of the isolates, *S.* Senftenberg 775W, *S.* Kedougou and *S.* Montevideo displayed ‘linear shoulder’ or ‘convex’ shapes [19]. Using the GInaFit modelling package it was found that these survivor curves could be predicted most accurately by models based on Weibull distributions of resistance. Both the modified Weibull model described by Albert and Mafart [22] and the double Weibull model described by Coroller et al. [23] fitted the data well. The delta values predicted by the modified Weibull models were higher than the DRT values derived from the slopes of the corresponding log-linear survivor plots (Table 3), reflecting the slower initial death rates for these isolates under these conditions. At 25 °C/15% RH, all isolates showed log-linear linear survival curves and retained significant viable numbers at 22 days (Figure 2). At 37 °C/20% RH, all isolates showed very rapid killing, with no survivors detected after one or two days of exposure under these conditions (Figure 3).

Temperature and RH together had a major impact upon the survival of the organisms. At 10 °C and 70% humidity the factory strain of *S.* Senftenberg demonstrated the greatest survival capacity (D_10_ = 6.1) compared to all other strains in the panel. High humidity (70%) coupled with low temperature resulted in a rapid decrease in cell counts. At 25 °C and 15% RH, *S*. Senftenberg 775W demonstrated the greatest survival compared to all other isolates (D_25_ = 18.04) followed by the factory isolate of *S*. Livingstone (D_25_ = 15.48) and *S*. Kedougou (D_25_ = 14.95). Of the panel of serotype-matched isolates, only the factory strain of *S.* Schwarzengrund (D_25_ = 9.1) demonstrated greater survival capacity compared to the clinical and veterinary strains. At 37 °C and 20% RH, a rapid decrease was observed with all strains dead within 48 h; a 7 log reduction was observed, equating to an average D_37_ = 6.48 h.

**Figure 4:**
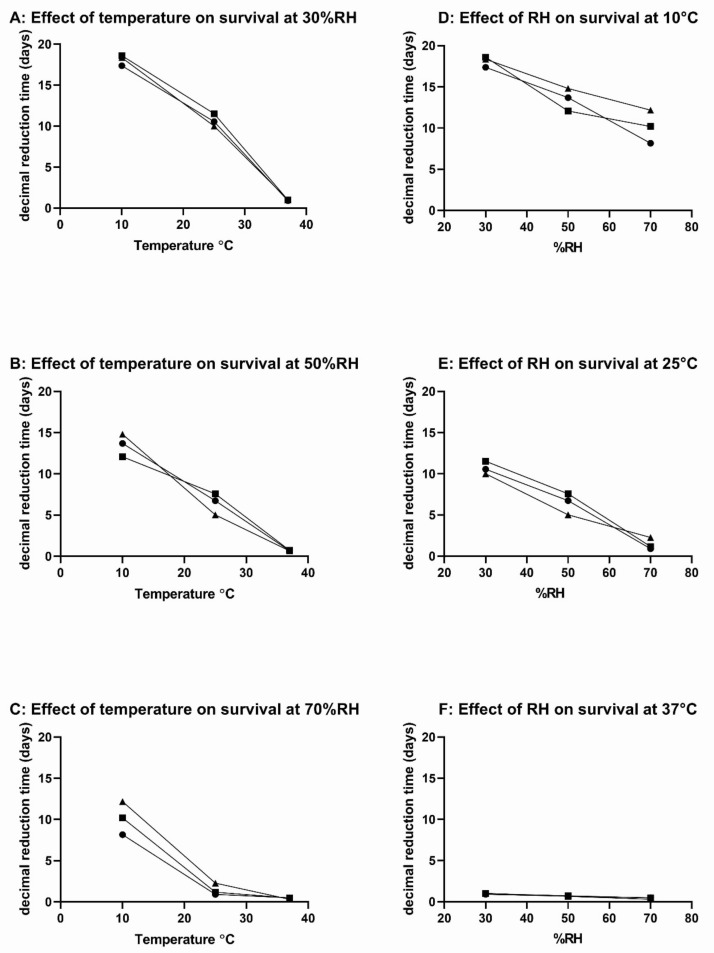
Effect of temperature (**A**–**C**) and RH (**D**–**F**) on survival of three serotype-matched *S*. Schwarzengrund isolates on stainless steel. Closed circles = clinical isolate; closed squares = factory isolate; closed triangles = veterinary isolate.

To explore further the interplay between temperature and RH on survival, the survival of three *S.* Schwarzengrund isolates at a combination of temperatures (10, 25 and 37 °C) and RH (30, 50 and 70%) was investigated. These isolates were selected as they displayed classical first order kinetics of survival on stainless steel coupons. The DRT values were calculated from the slopes of the survivor graphs. These were plotted as a function of temperature and RH in Figure 4. The DRT values for the three serotype-matched isolates decreased with increasing temperature at each RH studied. Similarly, the DRT values decreased with increasing RH at each temperature studied.

## 4. Discussion

Previous studies have highlighted contamination of factory surfaces with *Salmonella* as an important issue [3]. However, there is limited knowledge on the survival of resident factory *Salmonella* strains that have adapted to the environment in comparison to recent contaminants. In this study a panel of ten isolates consisting of factory, veterinary and clinical strains were compared in their survival ability on stainless steel coupons representing a widely used food manufacturing surface. Survival of these isolates was investigated under different combinations of temperature and RH encountered in food manufacturing environments. Significantly, it was shown that at 37 °C/20% RH, factory, veterinary and clinical strains were unable to survive more than 48 h. By contrast, at 25 °C/15% RH all isolates of *Salmonella* survived for over 22 days at levels between 10^3^ to 10^5^ from a starting population of 10^6^ to 10^7^. At 10 °C only the factory isolates of *S*. Schwarzengrund and *S*. Senftenberg and *S*. Typhimurium SL1344 retained viability levels around 10^3^–10^4^ cfu per coupon from starting innocula of 10^6^ cfu per coupon at 22 days. The factory strain of *S.* Senftenberg displayed the greatest survival capacity (D_10_ = 6.1), and the remainder of the factory strains did not exhibit any increased survival in comparison to strains from the other environments. Under most exposure conditions survivor curves were classified by Geeraerd et al. as ‘linear’ [19], indicating first order kinetics of inactivation from populations with uniform sensitivity to stress. The ‘linear shoulder’ or ‘convex’ survivor curves displayed by *S*. Senftenberg 775W, *S*. Kedougou and *S*. Montevideo at 10°C /70% RH indicate initial resistance to stress in these isolates under these conditions. This might reflect the accumulation of injury events that must occur before cell death [19,20,22].

Temperature plays an important role in *Salmonella* persistence on surfaces, elevating the temperature can cause cell damage and death due to the cellular components being destroyed. The factory strains of *Salmonella* as a group only showed enhanced survival at 25°C/15% RH compared to the other two groups of isolates. Chaitiemwong et al. investigated the survival of *Listeria monocytogenes* on a conveyor belt, using identical temperatures and similar humidity levels [24]. The study also revealed that survival was better at 25 °C in comparison to 37 °C and in line with the current study at 37 °C and 20% RH; results indicated a rapid decline in survival during the first 6 h. Moreover, at 10 °C and high humidity (70% RH) a rapid decrease in cell counts was also observed [24]. More recently, low temperature and RH have been shown to favour survival of *Staphylococcus aureus* and *Escherichia coli* on stainless steel surfaces [25].

*Salmonella* is able to persist in food environments for several years [4]. The survival of resident factory strains in comparison to others has not been widely investigated. The results of this study indicate that the factory strain of *S.* Schwarzengrund survived better than clinical and veterinary strains of the same serotype at 25 °C/15% RH and at 10 °C/70% the factory isolate of *S.* Senftenberg survived better than all the other isolates in the panel. Similarly, Habimana et al., compared the survival of resident strains in feed processing plants in response to stress factors typically found in the factory [14]. Their study reported that resident factory isolates survived better than other isolates in humid and dry conditions over a period of 28 days. They also found that *Salmonella* survival was better at 30 °C and 35% RH than 12 °C and 85% RH with *Salmonella* levels becoming undetectable after 28 days at the lower temperature.

Margas et al., employed an identical method to investigate the survival of a panel of *Salmonella* isolates on stainless steel at 23 °C/15% RH [17]. The highest survival (10^4−4.5^ cfu) was reported for *S.* Typhimurium DT104, *S.* Enteritidis and *S.* Agona with the average survival for *Salmonella* after 30 days being 10^2.5−3.5^ cfu. Interestingly, the study observed differences in survival within a serotype, with the highest and lowest survival being for isolates of *S.* Typhimurium. In line with these results, the study presented here showed an average 10^3−5^ cfu survival after 22 days at 25 °C/15% RH with factory isolates of both *S.* Schwarzengrund and *S.* Senftenberg exhibiting higher DRT values in comparison to serotype-matched clinical and veterinary isolates. In agreement with Margas et al. [17] this study found survivors at 25 °C/15% RH for all isolates at an independent reading 72 days (data not shown), indicating the possible existence of subpopulations with long-term survival characteristics present in each isolate. Furthermore, at 10 °C the factory isolate of *S.* Senftenberg displayed the longest D value on steel (D_10_ = 6.1) in comparison to serotype-matched clinical (D_10_ = 2.76) and veterinary (D_10_ = 3.17) isolates. This indicates that enhanced survival is not serotype specific.

This study shows that temperature and relative humidity independently influence the survival of *S.* Schwarzengrund isolates on stainless steel; increasing temperatures between 10 °C and 37 °C and increasing relative humidity levels from 30–70% both decrease the DRT values (Figure 4). Whilst lower temperatures are required for food handling, processing and packaging, higher temperatures would limit the survival of *Salmonella* on stainless steel surfaces. This highlights the need to control environmental conditions to limit the persistence of microbes in the food manufacturing environment, particularly during the factory shut-down period for cleaning when higher temperature/humidity levels could be introduced. It is possible that *Salmonella* survive better under temperatures outside the normal growth range reference range of temperature and relative humidity due to their dormant metabolic state. Under permissive conditions of temperature and relative humidity, it is possible that lack of nutrients leads to cell starvation, leading to decreased survival.

In terms of selection of resistance in response to environmental conditions, the increased resistance of the factory isolate at 25 °C/15% RH (Table 3) was not observed at the higher RH levels at 25°C (Figure 4). This suggests that lower humidity levels (15%) might select for isolates showing increased ability to survive. The factory *S.* Schwarzengrund was linked to human infections caused by contaminated dry dog and cat food in the United States [6,26]. Further examination showed that 79 people were affected, of which 48% were children under the age of two and illness in infants was associated with feeding pets in the kitchen. A case study was conducted to identify the cause of the outbreak and concluded that infection might have resulted from practices in a limited number of households. Suggesting that organisms multiplied in some households due to cross-contamination in the kitchens or pet food bowls not being thoroughly cleaned, both may support bacterial growth [6].

This study has concentrated on the survival of *Salmonella* on stainless steel work surfaces but is also relevant to the persistence and spoilage of other food products by *Salmonella*. Various studies have investigated these temperature and RH levels in a range of products. Interestingly, Deblais et al. found that temperature and relative humidity affected *Salmonella* invasion and survival in tomatoes and Lobacz et al. reported increasing temperature decreased the survival of *Salmonella* in cheese [27,28].

## 5. Conclusions

The persistence of *Salmonella* on contact surfaces is of great concern to the food industry as they may serve as a focus for cross-contamination of food products. Survival of all isolates depended upon the interaction of temperature and humidity rather than upon each factor in isolation. These parameters play a key role in determining the survival of *Salmonella* on stainless steel surfaces, with DRT values ranging from less than a day to over 18 days. There is some evidence to suggest that persistent food factory isolates show some mechanism of survival, particularly against temperature and relative humidity parameters and these should be addressed in the design and operation of food processing plants in order to limit the persistence and spread of pathogens.

## Figures and Tables

**Table 1:** Challenge panel of isolates.

Strain	Source
*S*. Schwarzengrund	Pet food factory USA
*S*. Schwarzengrund	FSL S5-458 American clinical
*S*. Schwarzengrund	Veterinary
*S*. Senftenberg	Pet food factory UK
*S*. Senftenberg 775W	ATCC 43845
*S.* Senftenberg	Veterinary
*S*. Livingstone	Pet food factory UK
*S*. Kedougou	Pet food factory UK
*S*. Montevideo	Pet food factory UK
*S*. Typhimurium SL1344	NCTC 13347

The names of the strains listed (Schwarzengrund, Senftenberg, Livingstone, Kedougou, Montevideo and Typhimurium) refer to the serotypes of *Salmonella enterica* subsp. *Enterica*.

**Table 2:** Temperature and RH conditions tested.

	15% RH	20% RH	30% RH	50% RH	70% RH
37 °C		X	X	X	X
25 °C	X		X	X	X
10 °C			X	X	X

X indicates the various combinations of RH and temperature studied. These were chosen to reflect environmental conditions measured in different regions of a manufacturing unit based upon monitoring data.

**Table 3:** Decimal reduction times (days) for *Salmonella* isolates.

Strains	DRT at 10 °C/70% RH	DRT at 25 °C/15% RH	DRT at 37 °C/20% RH
*S*. Schwarzengrund factory	4.91	9.12	0.26
*S*. Schwarzengrund clinical	3.90	8.08	0.26
*S*. Schwarzengrund vet	3.68	7.54	0.26
*S*. Senftenberg factory	6.10	10.68	0.27
*S.* Senftenberg 775W	2.76 (3.41) *	18.04	0.29
*S*. Senftenberg vet	3.17	9.52	0.26
*S*. Livingstone factory	3.54	15.48	0.29
*S*. Kedougou factory	3.13 (7.05) *	14.95	0.27
*S*. Montevideo factory	2.79 (5.20) *	8.94	0.27
*S*. Typhimurium SL1344	4.97	7.83	0.28

* Values in parentheses show the delta values (days) determined from modified Weibull models of bacterial inactivation [22] for isolates displaying non-log-linear inactivation.

## Data Availability

The parameters for modified Weibull models of non-linear survivor curves derived from Albert and Mafart [22] using GInaFiT [19] are provided in Supplementary Materials.

## Supplementary Materials

The following supporting information can be downloaded at: https://www.mdpi.com/article/10.3390/ijerph19020909/s1. Table S1: Parameters for modified Weibull models of non-linear survivor curves developed using GInaFiT.
