# Supplementary material for: Combined Effect of Temperature and Relative Humidity on the Survival of Salmonella Isolates on Stainless Steel Coupons
Source: Int J Environ Res Public Health. 2022 Jan 14;19(2):909. doi: 10.3390/ijerph19020909 (PMC8776188; doi:10.3390/ijerph19020909)
Supplement: Supplementary file 1 [file ijerph-19-00909-s001.zip › ijerph-1477457-SI.pdf]

Supplementary information Table S1

Parameters for modified Weibull models of non-linear survivor curves developed using GInaFiT (Geeraerd et al 2005).

Modified Weibull models derived from Albert and Mafart (2005) have the form:

N= (N0-Nres)10**(-((t/delta)**p))+ Nres

Where N is the bacterial count predicted by the model (cfu per coupon), N0 is the initial bacterial count (cfu per coupon), Nres is the residual bacterial count at the end of observation period (cfu per coupon), t is time (days), delta and p are model parameters.

This is reformulated in GInaFiT as:

LOG10(N)=LOG10((10**LOG10(N0)-10**LOG10(Nres))*10**((-t/delta)**p)+10**log10(Nres))

***S*. Senftenberg 775W at 10°C/70% RH**

parameter	value	Standard error		
LOG10(Nres)	0.00	0.00	Mean Sum of Squared Error	0.0000
delta	7.71	0.00	Root Mean Sum of Squared Error	0.0015
p	4.92	0.01	R-Square	1.0000
LOG10(N0)	6.38	0.00	R-Square adjusted	1.0000

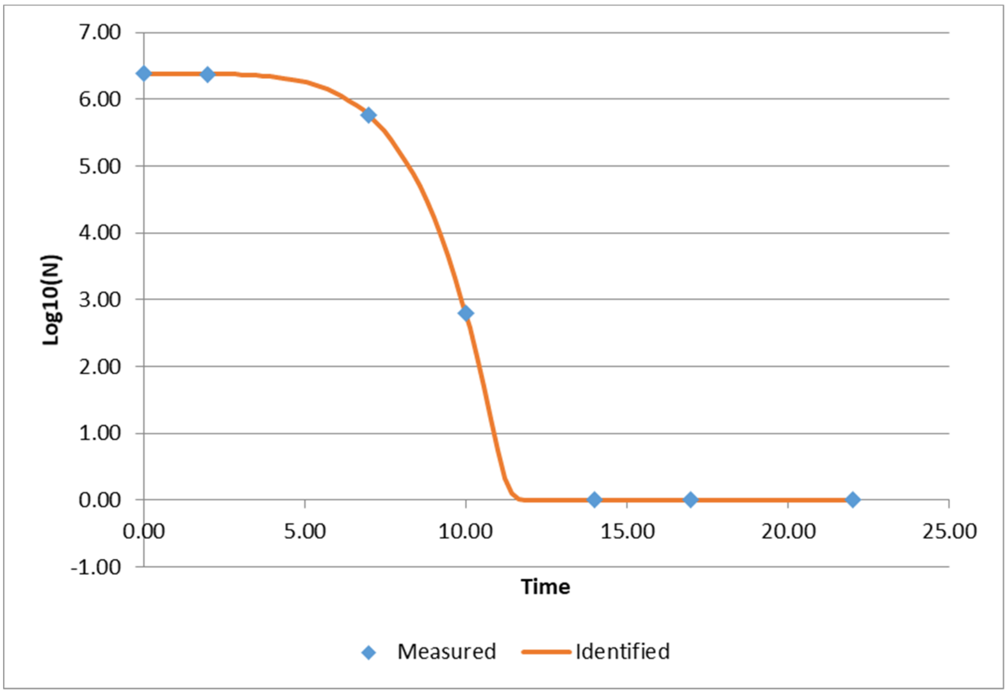

***S*. Kedougou factory at 10°C/70% RH**

parameter	value	Standard error		
LOG10(Nres)	-0.02	0.29	Mean Sum of Squared Error	0.0845
delta	10.77	0.76	Root Mean Sum of Squared Error	0.2906
p	3.79	0.58	R-Square	0.9943
LOG10(N0)	6.33	0.18	R-Square adjusted	0.9886

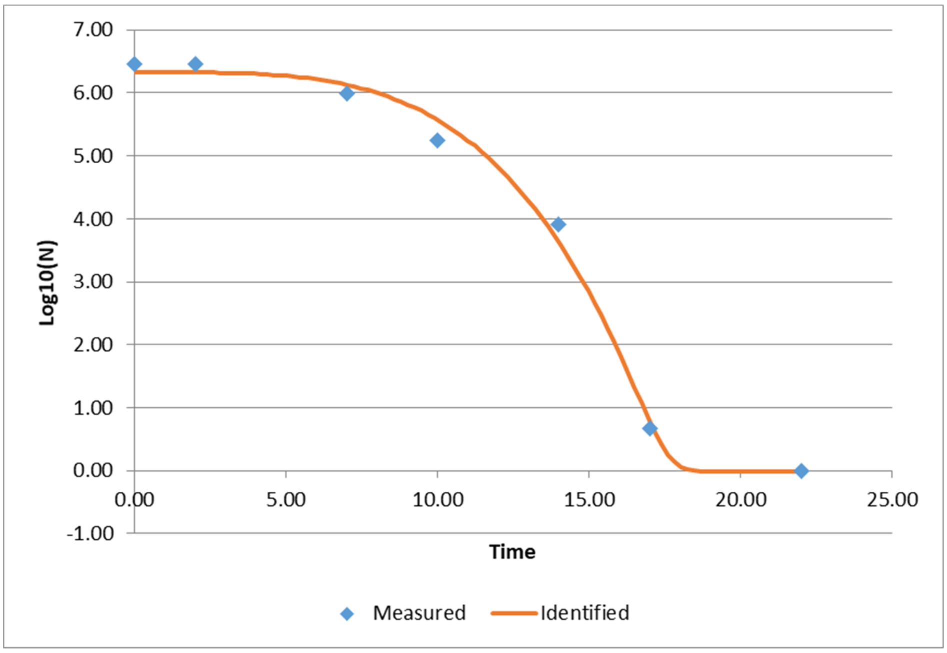

S. Montevideo factory at 10°C/70% RH

parameter	value	Standard error		
LOG10(Nres)	0.00	0.11	Mean Sum of Squared Error	0.0255
delta	10.23	0.44	Root Mean Sum of Squared Error	0.1597
p	4.74	0.61	R-Square	0.9987
LOG10(N0)	6.53	0.10	R-Square adjusted	0.9973

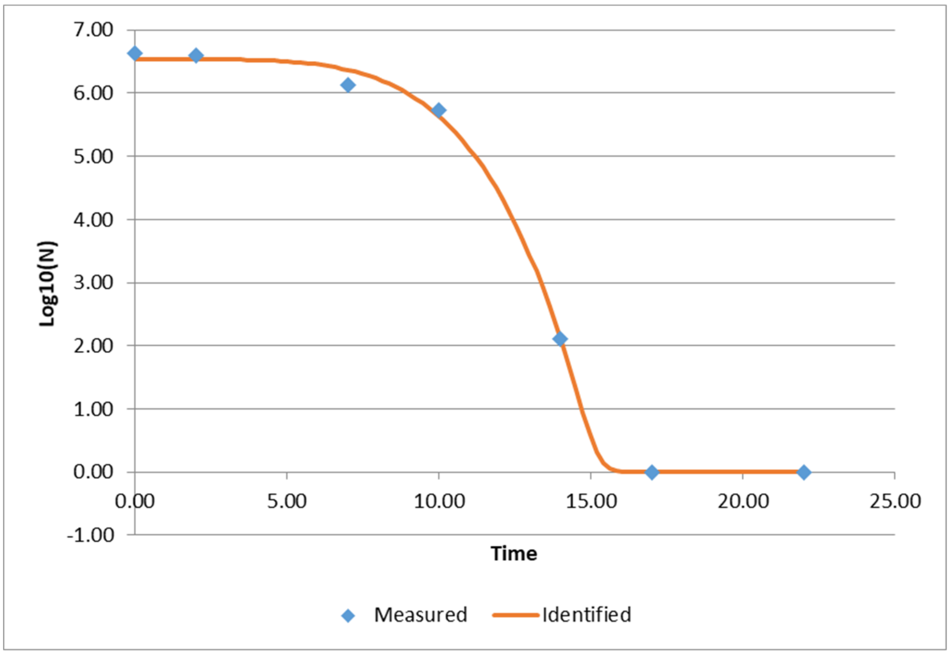

References

Albert, I.; Mafart, P.A. Modified Weibull model for bacterial inactivation. Int. J. Food Microbiol. 2005, 100, 197-211.

Geeraerd, A.; Valdramidis, V.; Van Impe, J. GInaFiT, a freeware tool to assess non-log-linear microbial survivor curves. Int. J. Food Microbiol. 2005, 102, 95-105.
